# Cholesterol-induced colorectal cancer progression and its mitigation through gut microbiota remodeling and simvastatin treatment

**DOI:** 10.1186/s12885-025-14379-3

**Published:** 2025-06-01

**Authors:** Xiaoliang Xie, Wenjing Wang, Haiming Zhang, Shaohui Zhao, Na Zhang, Ying Gao, Quanxia Liu, Xiaomei Chen

**Affiliations:** 1 Department of Colorectal Surgery, General Hospital of Ningxia Medical University, Yinchuan, 750004 China; 2 Ningxia Medical University, Yinchuan, 750004 China; 3 Department of Obstetrics and Gynecology, Ningxia Hui Autonomous Region People’s Hospital, Yinchuan, 750004 China; 4 Department of Oncology, People’s Hospital of Ningxia Hui Autonomous Region, Yinchuan, Ningxia China; 5 The Second Department of Oncology, Ningxia Medical University General Hospital, Yinchuan, 750004 China; 6 Department of Geriatrics, Hospital of Chengdu University of Traditional Chinese Medicine, Chengdu, China

**Keywords:** Colorectal cancer, Low-density lipoprotein, Gut microbiota, Simvastatin, *Lactobacillus*

## Abstract

**Background:**

Elevated serum cholesterol levels are linked to an increased risk of colorectal adenomas and colorectal cancer (CRC), yet the role of serum low-density lipoprotein (LDL) in CRC development remains unclear. This study explores the impact of cholesterol on tumor growth and the potential therapeutic effects of *Lactobacillus* and Simvastatin.

**Methods:**

We utilized a cecal tumor xenograft mouse model with Ldlr^−/−^ mice to assess the effects of high cholesterol levels on tumor growth. Additionally, the role of gut microbiota remodeling and cholesterol-lowering strategies was investigated using *Lactobacillus* supplementation and Simvastatin treatment.

**Results:**

Ldlr^−/−^ mice on a high-cholesterol diet developed significantly larger tumors (*P* < 0.05) and exhibited exacerbated malignancy, as indicated by HE and Ki-67 staining. *Lactobacillus* supplementation reduced tumor growth (*P* < 0.05), lowered serum cholesterol levels, and altered the gut microbiota composition, increasing the relative abundance of beneficial bacterial taxa. Simvastatin treatment reduced PD-L1 expression in CRC cells by lowering cholesterol levels, which was associated with decreased CRC proliferation, reduced serum LDL levels, and enhanced T cell infiltration in the tumor microenvironment.

**Conclusion:**

Elevated serum cholesterol promotes CRC progression, while gut microbiota remodeling through *Lactobacillus* supplementation and cholesterol-lowering interventions, such as Simvastatin, show potential in mitigating tumor growth and enhancing antitumor immune responses. These findings highlight the importance of cholesterol management in CRC treatment strategies.

**Supplementary Information:**

The online version contains supplementary material available at 10.1186/s12885-025-14379-3.

## Introduction

Cancer cells require more resources (such as glucose, glutamine, and other amino acids) for survival and macromolecular production due to metabolic difficulties in the tumor microenvironment. Malignant cells produce more fatty acids (FA), which provide energy through β-oxidation and can be converted to triglycerides or phospholipids for membrane formation [1, 2]. Cholesterol is a neutral lipid that is necessary for membrane integrity and fluidity [3]. Its primary purpose is to keep the cell membrane stable and regulate its fluidity. Thus, cholesterol plays a critical role in membrane production and proliferation during tumor formation [4]. Cholesterol and low-density lipoprotein receptor (LDLR) expression are both risk factors and drivers of tumor growth. Cells take up cholesterol from circulating LDL via the cell surface LDLR [5] and continue to proliferate rapidly. The mammalian gut can react to nutritional signals. Diet is becoming recognized as a significant risk factor for cancer formation and progression in a range of tumor forms, with higher incidences of gastric, lung, renal, breast, and colon/rectal malignancies [6]. According to current research, cholesterol is linked in the evolution of breast cancer [7], bladder cancer [8], colorectal cancer (CRC) [9], and CRC-related hepatic metastases [10]. However, previous mouse models used to study the role of cholesterol in CRC progression have primarily used immunocompetent or immunodeficient mice on a high cholesterol diet, and in humans with elevated cholesterol, the main components are LDL and VLDL cholesterol [11]. The mechanisms by which cholesterol contributes to CRC progression via LDLR uptake are not fully understood. Cholesterol is a structural component of the plasma membrane that modulates membrane fluidity, solubilizes other lipids, and functions as a signaling mediator [12]. Thus, cholesterol metabolism regulates anti-tumor immune responses by interacting with a diverse variety of immune cells engaged in both innate and adaptive immune responses. For example, high cholesterol metabolism-associated macrophage morphology is linked to poor survival in colorectal cancer patients [13]. Cholesterol may stimulate the progression of CRC by activating the PI3K/AKT signaling pathway; however, cholesterol may not affect the number of tumors formed in CRC [14]. In addition, cholesterol was discovered to mainly affect the advanced stages of CRC rather than the early stages [14]. Notably, cholesterol in the tumor microenvironment (TME) has been demonstrated to induce CD8(+) T cell “depletion” in a mouse melanoma model, suggesting cholesterol’s function in suppressing anti-tumor immune responses [15]. In mice, high serum cholesterol levels improve the anti-tumor action of natural killer cells and inhibit liver tumor growth [16]. However, evidence correlating high cholesterol with immune response in CRC is still lacking.

Probiotics and commensal bacteria have been shown to alter intestinal flora structure, improve mucosal barrier function [17], reduce blood cholesterol levels [18], and stimulate the host immune system [19, 20]. Previous research has indicated that probiotics, such as *Lactobacillus* and *Bifidobacterium*, can help reduce inflammatory bowel disease (IBD) and colorectal carcinogenesis in mouse models [21, 22]. Recent research indicates that *Lactobacillus* plantarum-derived indole-3-lactic acid reduces colorectal carcinogenesis via epigenetic control of CD8(+) T cell immunity [23]. However, the relationship between cholesterol and *Lactobacillus* and intestinal commensals is not well understood.

Here is the polished translation adhering to scientific conventions and ensuring low text similarity: Our study demonstrates that high-fat diet markedly increases colorectal tumor burden in Ldlr^−/−^ mice accompanied by elevated serum LDL/VLDL levels. Mechanistic investigations revealed that probiotic administration not only counteracts LDLR deficiency-driven colorectal carcinogenesis through gut microbiota remodeling, but also suppresses programmed death-ligand 1 (PD-L1) expression via cholesterol metabolic regulation, thereby reprogramming the tumor immune microenvironment. These findings propose novel therapeutic strategies for combinatorial immunotherapy and adjuvant interventions in CRC.

## Methods

### Experimental animal models

WT C57BL/6J mice and Ldlr^−/−^ mice were purchased from Collective Pharmacare (GemPharmatech, China) and housed under specific pathogen free (SPF) conditions. A mouse in situ colon cancer model was established by dispersing 1 × 10^6^ luciferase-expressing MC38 cells (MC38-luc) in ~ 40 µL of PBS and injecting them directly into the cecum of C57 mice. The first part consisted of 4 groups: intestinal dysbiosis was induced by antibiotics according to the previous method [24]. (1) model mice: lactobacilli (gavage, 0.66 g/day; LeTuoEr, France) + regular diet; (2) model mice: regular diet + saline (gavage, 0.2 mL); (3) model mice: antibiotic depletion + regular diet + saline (gavage, 0.2 mL); (4) model mice: antibiotic depletion + lactobacilli (gavage, 0.66 g /day; LeTuoEr, France) + regular diet. In the antibiotic depletion group, the model mice were given 0.02 mL/g ceftriaxone intragastric administration every two days, and the concentration of ceftriaxone sodium was 0.6 g/mL (MCE, HY-B0712). The second part consisted of 2 groups: simvastatin dissolved in water and mice were orally administered simvastatin (4 mg/kg) daily. (1) model mice: high cholesterol diet; (2) model mice: high cholesterol diet + oral simvastatin.

Male and female animals of 8 weeks of age were used in our study. All the mice were euthanized with an intraperitoneal injection of pentobarbital sodium (2% solution was prepared with sterile saline), and the tumors were excised for analysis and weighting. Fasting serum, feces, and intestines were retained at the end of the experiment for further study. All experiments were conducted in accordance with institutional and ethical guidelines and approved by the Animal Care and Use Committee.

### Hematoxylin-eosin (H&E) stain and immunohistochemical staining

Colonic tissues were collected from experimental animal models for H&E staining. Briefly, colon tissues were fixed in formalin, embedded in paraffin and cut into 5 μm slices. After staining with H&E, the samples were visualized for pathological changes using a light microscope.

For immunohistochemical saturate buffer for 15 min. Non-specific antigens were blocked with 5% goat serum for 30 min. The following antibody information was used in this study: Ki67 (1:100, ab833, Abcam); PDL1 (1:1000, ab205921, Abcam) CD3 (1:150, ab16669, Abcam). The secondary biotinylated coupled goat anti-rabbit antibody was incubated for 30 min at room temperature. DAB was used as a chromogen and then stained with hematoxylin and blocked with dimethylphenyl blocker.

### Detection of biochemical indicators in serum

Serum was collected from experimental animals and an ELISA kit was used to determine mouse low-density lipoprotein cholesterol (LDL-C) (#MBS748297); interleukin- 2 (IL-2) (ab100706; Abcam); tumor necrosis factor-α (TNF-α) (ab46105; Abcam); and TNF-β (ab100689; Abcam). It was measured at 450 nm on an enzyme labeling instrument (BioTek).

### Cell line experiments

The CRC cell line used in this paper was SW480 and HT-29, which was obtained and characterized by the Cell Bank of the Chinese Academy of Sciences (Shanghai, China). It was cultured in Duchenne modified Eagle medium (DMEM) containing 10% fetal bovine serum (FBS) and 1% penicillin and streptomycin. Cell culture conditions were 37 °C with 5% CO_2_ humidified air. Cholesterol and simvastatin were purchased from Sigma-Aldrich. different concentrations of cholesterol (0 ~ 50 μm), simvastatin (0 ~ 10 μm) was added as treatments to detect PD-L1 expression.

### Western blot

Cells were digested with trypsin and collected in 1.5 ml EP tubes in RIPA buffer supplemented with protease inhibitors for 15 min on ice before centrifugation at 12,000 × g for 20 min at 4 °C. Equal amounts of proteins from the entire lysates were resolved by 15% SDS-PAGE, and the separated proteins were transferred to a nitrocellulose membrane (Bio-Rad Laboratories, Inc., Hercules, CA, USA) with Tris-glycine buffer. Blots were blocked in blocking buffer (5% skimmed milk in TBST) for 1.5 h at room temperature before incubating with primary antibodies overnight at 4 °C. Following washing, the membranes were incubated for 1 h with anti-mouse or anti-rabbit Ig-HRP and identified using the ECL system. The optical density was calculated using ImageJ software (USA).

### Real-time quantitative polymerase chain reaction (RT-qPCR)

RT-qPCR was performed to reveal levels of *Lactobacillus*. Total DNA was isolated from patient and mouse fecal pellets using the QIAamp Fast DNA Fecal Mini Kit (Qiagen) according to the manufacturer’s protocol.The RT-qPCR primers used in this experiment were as follows: *Lactobacillus*.

forward (5’-3’) TGGAAACAGRTGCTAATACCG.

reverse (5’-3’) GTCCATTGTGGAAGATTCCC.

### 16 S rRNA sequencing

16 S rRNA gene amplicon sequencing was performed by Applied Protein Technology Shanghai (APTBIO, Shanghai, China). DNA extraction, amplification, and library construction were performed with reference to the previous protocol [25]. The instrument Illumina NovaSeq 6000 was used, and the sequencing strategy was PE250 double-end sequencing for 16 S amplicon sequencing. The corresponding species information and species-based abundance distribution were obtained using the Qiime 2 analysis process [26].

### Statistical analysis

Data are expressed as mean ± SEM unless otherwise stated. Statistical analyses were performed using a two-tailed unpaired Student’s t-test to compare the two groups of interest, and *P* < 0.05 was considered statistically significant. Images were drawn using GraphPad Prism software.

## Results

### Elevated serum cholesterol promotes tumor growth

**Fig. 1:**
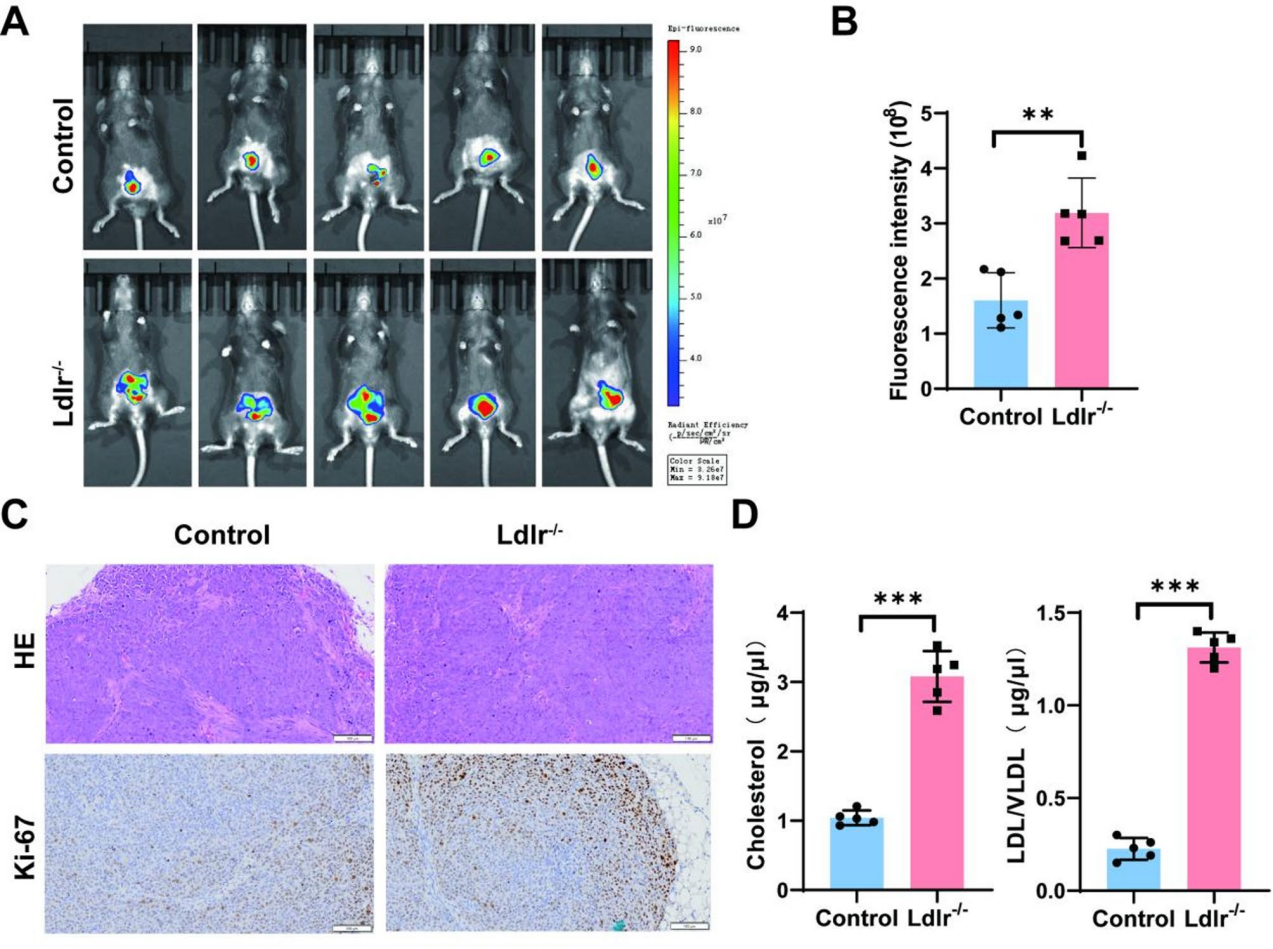
The role of Ldlr^−/−^ mice in CRC progression. (**A**) In vivo bioluminescence imaging analysis using MC38 cells expressing luciferase (MC38-Luc). (**B**) Quantification of bioluminescence intensity for each mouse, with the average value calculated for each cohort. Data are presented as mean ± SEM (*n* = 5). (**C**) Colonic tissues from Ldlr^−/−^ mice and controls are shown in HE staining and Ki67 staining. (**D**) Serum cholesterol and LDL/VLDL levels (µg/µl)

Increased serum cholesterol levels are associated with a higher risk of colorectal adenomas and CRC [27]. However, evidence for the role of serum low-density lipoprotein (LDL) in CRC development is still lacking. A high-cholesterol culture in a cecal tumor xenograft mouse model confirmed that Ldlr^−/−^ mice formed larger tumors (*P* < 0.05; Fig. 1A-B). Tumor weight data were also recorded, shown in Figure S1A, support the conclusion that Simvastatin treatment reduced tumor growth. Compared to the control group, both HE staining and Ki-67 staining indicated exacerbated malignant tumor growth (Fig. 1C). Additionally, the deficiency of LDLR significantly elevated serum cholesterol and LDL/VLDL levels (*P* < 0.05; Fig. 1D).

### *Lactobacillus* altered the ecological diversity of the fecal Microbiome

**Fig. 2:**
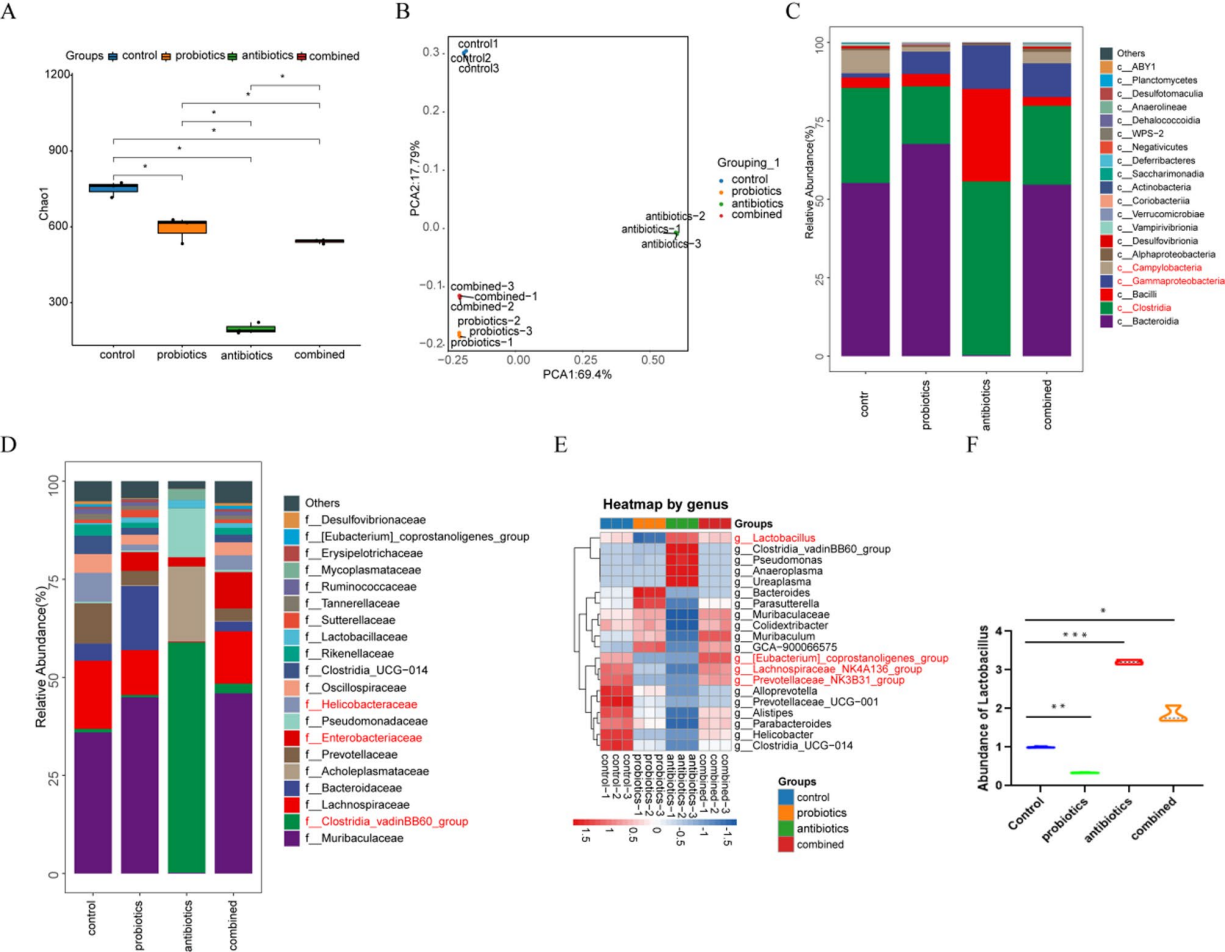
Analysis of microbiome diversity at different taxonomic levels. (**A**) Chao1 index at the OTU level for each group; (**B**) PCA model summarizing sample distribution; (**C**) Community analysis at the class level; (**D**) Community analysis at the family level; (**E**) Genus-level analysis of significantly different species across groups; (**F**) qPCR analysis of *Lactobacillus* in fecal samples from different groups. (*n* = 5; *, *P* < 0.05, **, *P* < 0.01, ***, *P* < 0.001) (OTU: operational taxonomic unit)

We sought to analyze the impact of *Lactobacillus* on the composition of the fecal microbiome to explore the extent of microbiome alterations under different treatments. Fecal samples were subjected to 16 S rRNA sequencing. Figure 2A shows that the Chao1 species richness index revealed significant differences in alpha diversity among the treatment groups (*P* < 0.05). Principal Component Analysis (PCA) based on 2269 OTUs demonstrated clear distinctions in gut microbiota composition between groups (Fig. 2B). To examine specific microbial changes in Ldlr^−/−^ mice, we assessed the relative abundance of taxa across different levels. At the class level, the proportions of Campylobacteria, Gammaproteobacteria, and Clostridia significantly increased following *Lactobacillus* supplementation compared to the antibiotic-only treatment group (Fig. 2C). At the family level, Helicobacteraceae, Enterobacteriaceae, and the Clostridia_vadinBB60_group were significantly enriched (Fig. 2D). At the genus level, we observed a decreasing trend in *Lactobacillus* abundance and an increasing trend in Bacteroides and Parasutterella following antibiotic treatment (Fig. 2E). After *Lactobacillus* supplementation, the [Eubacterium] coprostanoligenes group and Muribaculum were significantly increased (Fig. 2E). Additionally, we verified the levels of *Lactobacillus* in fecal samples through qPCR, confirming differences in *Lactobacillus* abundance across the treatment groups (Fig. 2F).

### Remodeling of the gut microbiota alleviates CRC tumor growth by reducing serum cholesterol levels

**Fig. 3:**
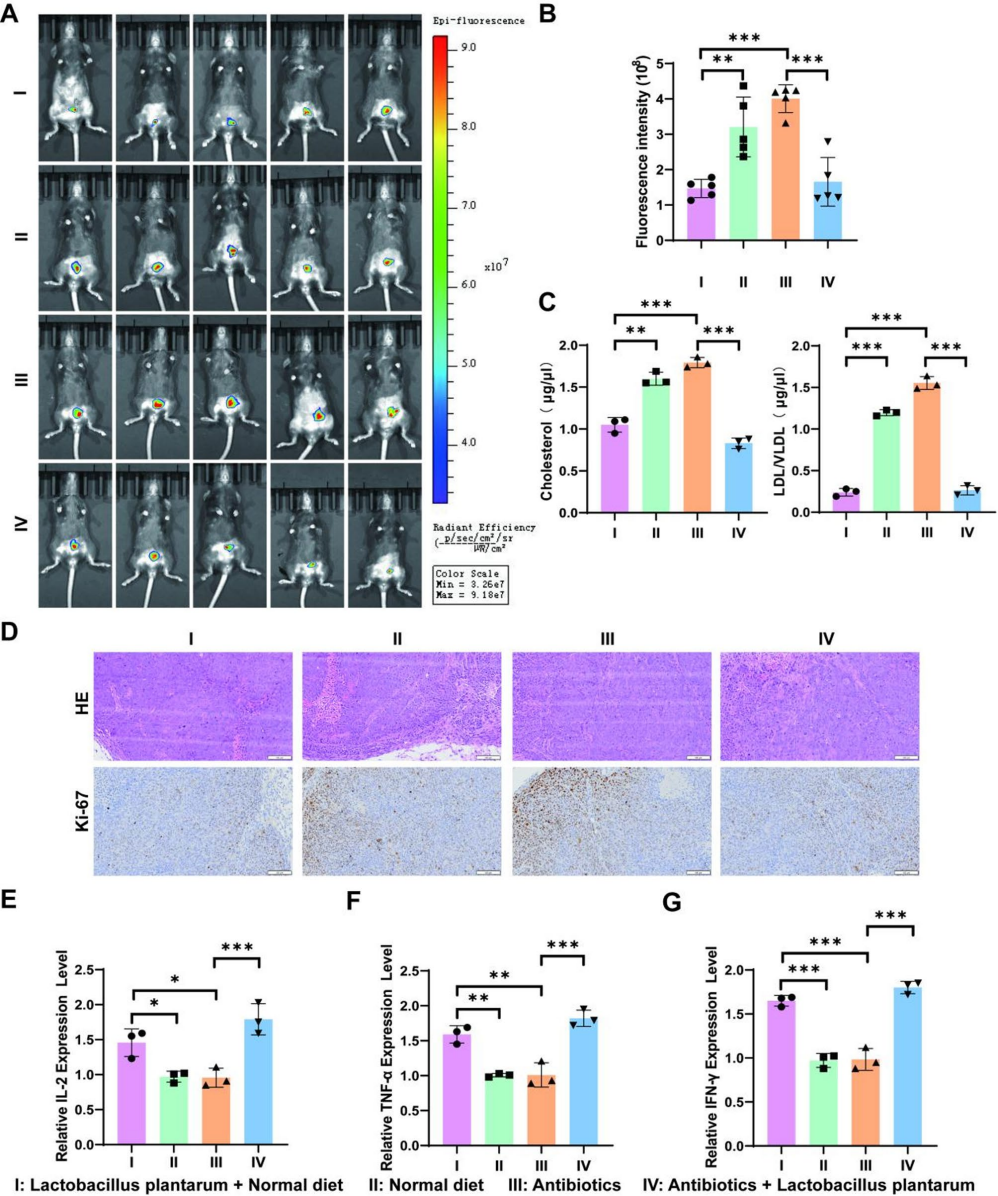
*Lactobacillus* plantarum-mediated reduction of serum cholesterol alleviates CRC tumor growth. (**A**) In vivo bioluminescence imaging showing tumor progression in four groups: I (Lactobacillus plantarum + Normal diet), II (Normal diet), III (Antibiotics), and IV (Antibiotics + Lactobacillus plantarum). Antibiotics group: 0.02mL/g ceftriaxone intragastric administration every two days. (**B**) Quantification of fluorescence intensity representing tumor size across the four groups. (**C**) Serum cholesterol and LDL/VLDL expression levels (µg/µl), showing a significant reduction with *Lactobacillus* plantarum supplementation. (**D**) Histological analysis of colon tissues from each group, with HE staining showing tissue architecture and Ki-67 staining highlighting proliferating cells. (**E**-**G**) Relative expression levels of inflammatory markers IL-2, TNF-α, and IFN-γ, respectively, in colon tissues across the four groups. Data are presented as mean ± SEM, with significant differences indicated (*,*P* < 0.05; **, *P* < 0.01; ***, *P* < 0.001)

Probiotics are defined as ‘dietary supplements containing live, non-pathogenic microorganisms or their derivatives that are beneficial to the host.’ The anticancer properties of probiotics have been confirmed, and their positive effects in treatment are evident. Several strains of *Lactobacillus* have been shown to effectively reduce cancer incidence [28, 29]. We sought to investigate whether *Lactobacillus* could alleviate tumor growth by lowering serum cholesterol levels. In a primary colorectal cancer model, we found that antibiotic treatment led to tumor enlargement, while *Lactobacillus* feeding significantly alleviated tumor growth (*P* < 0.05; Fig. 3A-B). Tumor weight data were also recorded, shown in Figure S1B, which support the conclusion that *Lactobacillus* plantarum supplementation reduced tumor growth. Furthermore, serum cholesterol and LDL/VLDL levels were significantly reduced with *Lactobacillus* supplementation (Fig. 3C). Correspondingly, HE staining and Ki-67 staining of colon tissue indicated that *Lactobacillus* supplementation mitigated CRC growth, whereas antibiotic treatment had the opposite effect (Fig. 3D).

### Simvastatin regulates PD-L1 expression in CRC by Lowering cholesterol

**Fig. 4:**
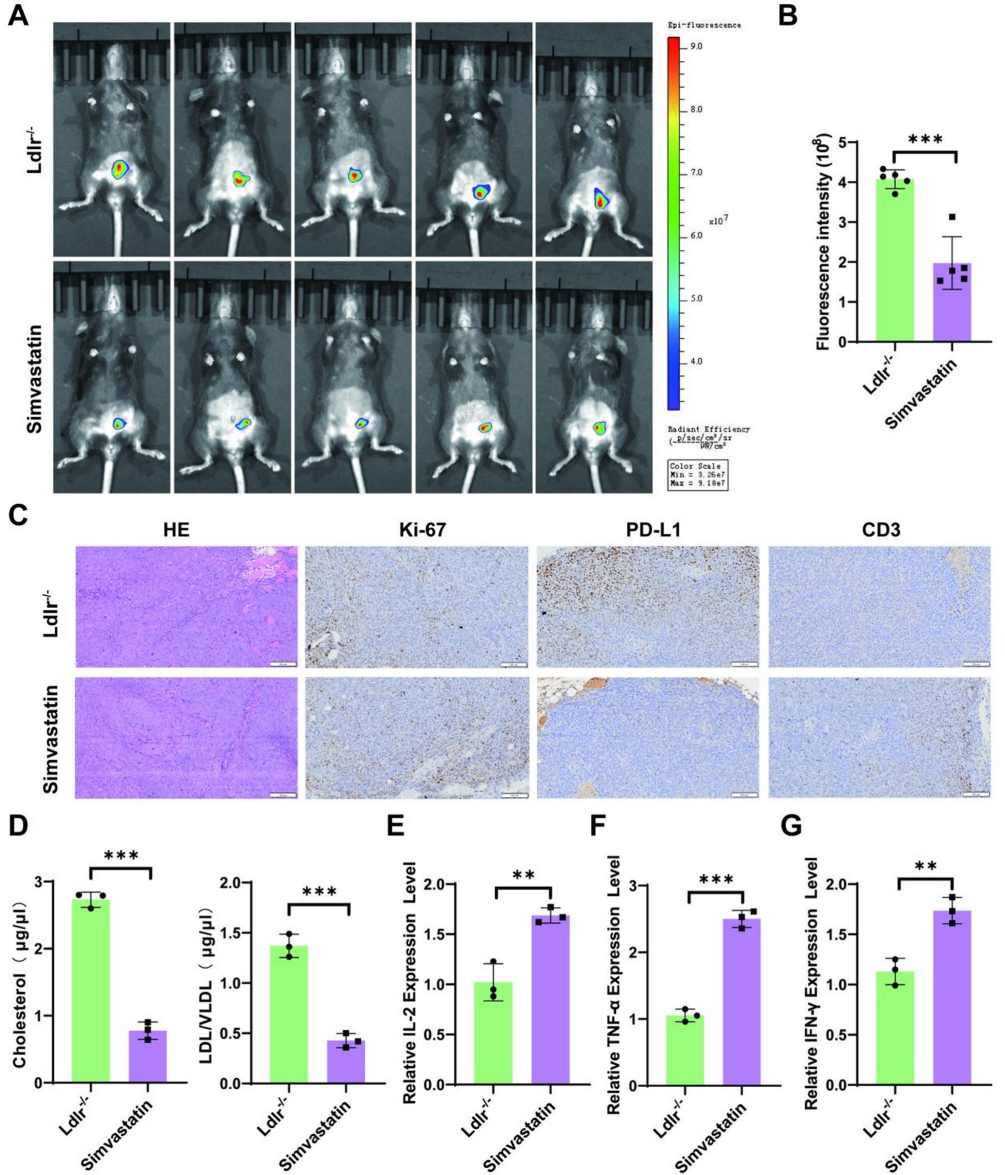
In Ldlr^−/−^ cecal xenograft mice simvastatin regulates PD-L1 expression in CRC by reducing cholesterol levels. (**A**) In vivo bioluminescence imaging after simvastatin treatment reveals tumor progression. (**B**) Quantification of bioluminescence intensity for each mouse, with the average value calculated for each cohort. Data are presented as mean ± SEM (*n* = 5). (**C**) Simvastatin-treated colon tissue HE, Ki67 staining, PD-L1 and CD3 staining. Serum levels of (**D**) Cholesterol and LDL/VLDL, (**E**) IL2, (**F**) TNF-α, and (**G**) TNF-γ were measured

We aimed to determine whether Simvastatin regulates PD-L1 expression in CRC through cholesterol reduction. In Ldlr^−/−^ cecal xenograft mice treated with Simvastatin, we observed a significant reduction in the bioluminescence signal of MC38-luc cells (*P* < 0.05; Fig. 4A-B). Tumor weight data were also recorded, shown in Figure S1E-F, support the conclusion that Simvastatin treatment reduced tumor growth. HE and Ki-67 staining indicated a slowdown in CRC proliferation. PD-L1 expression was reduced, accompanied by increased CD3 expression, suggesting enhanced T cell infiltration in the tumor microenvironment (Fig. 4C). Additionally, serum cholesterol and LDL/VLDL levels were significantly reduced (*P* < 0.05; Fig. 4D). Levels of IL-2, TNF-α, and IFN-γ decreased following Simvastatin treatment, which may indicate immune system activation and an enhanced antitumor response (*P* < 0.05; Fig. 4E-G).

### Cholesterol maintains PD-L1 stability in CRCs

**Fig. 5:**
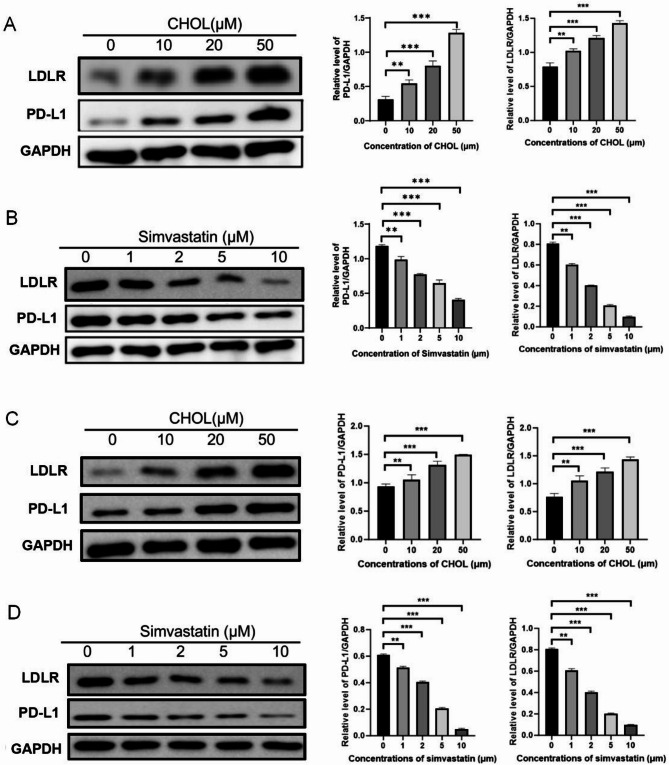
Cholesterol Maintains PD-L1 Stability in SW480 and HT-29. (**A**) Treatment with several concentrations of cholesterol (CHOL, 0 to 50 µM for 12 h) in SW480, (**B**) or simvastatin (0 to 10 µM for 12 h). Protein blotting was performed to measure the PD-L1 table after each treatment. PD-L1 expression intensity (relative to GAPDH) was quantified using ImageJ. (**C**) Treatment with several concentrations of cholesterol (CHOL, 0 to 50 µM for 12 h) in HT-29, (**D**) or simvastatin (0 to 10 µM for 12 h). Protein blotting was performed to measure the PD-L1 table after each treatment. PD-L1 expression intensity (relative to GAPDH) was quantified using ImageJ. Data shown are mean ± SD (*n* = 3)

To investigate the relationship between cholesterol and immunosuppression, we examined endogenous PD-L1 expression in cholesterol simvastatin-treated SW480 cells and HT-29 cells. Simvastatin is an FDA-approved drug for lowering total cholesterol [29]. Protein blot analysis showed that the addition of cholesterol to SW480 and HT-29 cells led to a dose-dependent upregulation of PD-L1 levels (Fig. 5A, C), whereas the addition of simvastatin downregulated the cellular abundance of PD-L1 with increasing dose (Fig. 5B, D). The trends observed in both cell lines were consistent, supporting a similar mechanism.

## Discussion

Our data indicate that in an immunocompetent model of hypercholesterolemia, LDLR impairment causes elevated blood LDL concentrations and subsequent CRC development. Furthermore, *Lactobacillus* treatment modified the intestinal milieu and lowered serum LDL levels, which helped to prevent CRC. Furthermore, our findings indicate that Simvastatin medication was successful in suppressing LDLR-mediated tumor growth in the context of hypercholesterolemia, with lower levels of PD-L1 expression and higher levels of CD3. Overall, these findings suggest that Simvastatin slows CRC growth by lowering circulating LDL levels, engaging the immune system, and increasing anti-tumor responses.

LDLR is primarily responsible for the endocytosis of cholesterol-rich LDL, with 70% of this process occurring in the liver [30]. Under normal conditions, LDLR is located within clathrin-coated pits. LDLR rapidly cycles between the cell surface and the interior, enabling cells to utilize serum cholesterol [31]. Due to its physiological function, LDLR has an unavoidable impact on intracellular and serum cholesterol metabolism. Emerging evidence suggests that hypercholesterolemia is associated with the growth of tumor metastases [32]. A high-cholesterol diet has been shown to increase the tumor burden of liver metastases from melanoma cells [33]. In CRC, existing studies have indicated that higher levels of LDLR expression are associated with advanced N and M stages of CRC. In vitro, LDL promotes CRC cell migration and spheroid formation [9], while in vivo, a high-cholesterol diet significantly shortens overall survival in mice with CRC liver metastases [10]. Previous studies have reported the direct role of LDL/HDL cholesterol in regulating colon cancer tumogenesis [34, 35]. Emerging evidence demostrates that decreased LDLR expression in cancer cells impairs LDL uptake but promotes HCC cell proliferation and metastasis in vivo and in vitro, potentially via the activation of the MEK/ERK signaling pathway to enhance de novo cholesterol synthesis [36]. Consequently, we preliminarily conclude that LDLR deficiency may induce increased de novo cholesterol synthesis in CRC, leading to poor survival outcomes.

In the tumor microenvironment, cholesterol metabolism is altered, resulting in immune system dysregulation, immune evasion, and tumor survival. It has been found that any change in cholesterol homeostasis can lead to an increased inflammatory response [37]. Furthermore, lipid accumulation in myeloid-derived suppressor cells (MDSCs) and tumor-associated macrophages (TAMs) converts these immune cells into immunosuppressive and anti-inflammatory phenotypes [38, 39]. T cells, which are critical participants in adaptive immunity, frequently lose function during tumor infiltration, and T cell exhaustion is a common occurrence in tumor progression. Therefore, there is an urgent need for therapies to restore T cell function [40]. Given the prevalence of cholesterol in tumors, lowering it can boost T cell activation [15, 41]. Our work shows that Simvastatin treatment helps reduce cholesterol-mediated PD-L1 levels and increase CD3 levels, providing further evidence that targeting cholesterol levels can enhance T cell activity.

Furthermore, disruption of cholesterol homeostasis via gut flora may be a risk factor for cancers that rely predominantly on cholesterol turnover. Our findings show that probiotic-mediated cholesterol reduction could be one potential approach for CRC treatment. Although our current study primarily focuses on compositional changes in the microbiota, prior research suggests that Lactobacillus supplementation influences cholesterol metabolism through mechanisms such as bile salt hydrolase activity and short-chain fatty acid production [40–41]. While functional analyses, such as microbial metabolomics or pathway activity assessments, would provide deeper insights, our data already demonstrate a clear correlation between Lactobacillus-induced microbiome remodeling, cholesterol reduction, and tumor inhibition. These findings suggest that dietary modifications may impact gut microbial composition, thus opening new avenues for diet control and/or targeting the gut microbiota as a potential cancer treatment.

We acknowledge that the safety and potential adverse effects of Lactobacillus supplementation and Simvastatin treatment, particularly for long-term use, were not addressed in our study. While previous clinical and preclinical data indicate that both Lactobacillus supplementation and statins are generally safe at appropriate doses [42–43], we agree that long-term toxicity data would be critical for the translational potential of these interventions. In our experiments, we did not observe overt toxicity, as the doses used were within physiologically relevant ranges. However, future studies should include long-term safety evaluations to assess the potential side effects of these interventions. We will revise the discussion to address this limitation and emphasize the importance of further toxicity and safety research for clinical translation.Furthermore, in this study, only free cholesterol treatment was used to detect the expressions of PD-L1 and LDLR. Subsequently, LDL/VLDL treatment will be further used to detect the expression levels of both. And the levels of cholesterol and LDL/VLDL in the serum of mice before tumor implantation were not detected in this study. More in-depth research is needed in the future to further prove that Lactobacillus regulates cholesterol metabolism and thereby inhibits the progression of CRC.

In conclusion, dysregulation of cholesterol homeostasis is critical for cancer development, and our work opens up new avenues for treating CRC through gut microenvironment remodeling, as well as providing strong evidence that serum cholesterol inhibition activates T-cell immunity to cure CRC. Further research into the involvement of cholesterol in cancer will provide more information and approaches for tumor control.

## Electronic supplementary material

Below is the link to the electronic supplementary material.

Supplementary Material 1: Figure S1: Tumor weight data for the animal experiments. (A) Tumor weight for the control and Ldlr-/- groups from Figure 1. (B) Tumor weight for the four groups of animals from Figure 2: I (Lactobacillus plantarum + Normal diet), II (Normal diet), III (Antibiotics), and IV (Antibiotics + Lactobacillus plantarum). (C) Tumor weight for the Ldlr-/- and Simvastatin treatment groups from Figure 5.

Supplementary Material 2

## Data Availability

The data that support the findings of this study are available from the corresponding author, [XC], upon reasonable request. The datasets generated and/or analysed during the current study are available in the [BioProject] repository, [https://www.ncbi.nlm.nih.gov/bioproject/?term=PRJNA1185438].
