# Supplementary material for: Cholesterol-induced colorectal cancer progression and its mitigation through gut microbiota remodeling and simvastatin treatment
Source: BMC Cancer. 2025 Jun 1;25:977. doi: 10.1186/s12885-025-14379-3 (PMC12128496; doi:10.1186/s12885-025-14379-3)
Supplement: Supplementary file 1 — Supplementary Material 1: Figure S1: Tumor weight data for the animal experiments. (A) Tumor weight for the control and Ldlr-/- groups from Figure 1. (B) Tumor weight for the four groups of animals from Figure 2: I (Lactobacillus plantarum + Normal diet), II (Normal diet), III (Antibiotics), and IV (Antibiotics + Lactobacillus plantarum). (C) Tumor weight for the Ldlr-/- and Simvastatin treatment groups from Figure 5. [file 12885_2025_14379_MOESM1_ESM.docx]

Figure 4 A


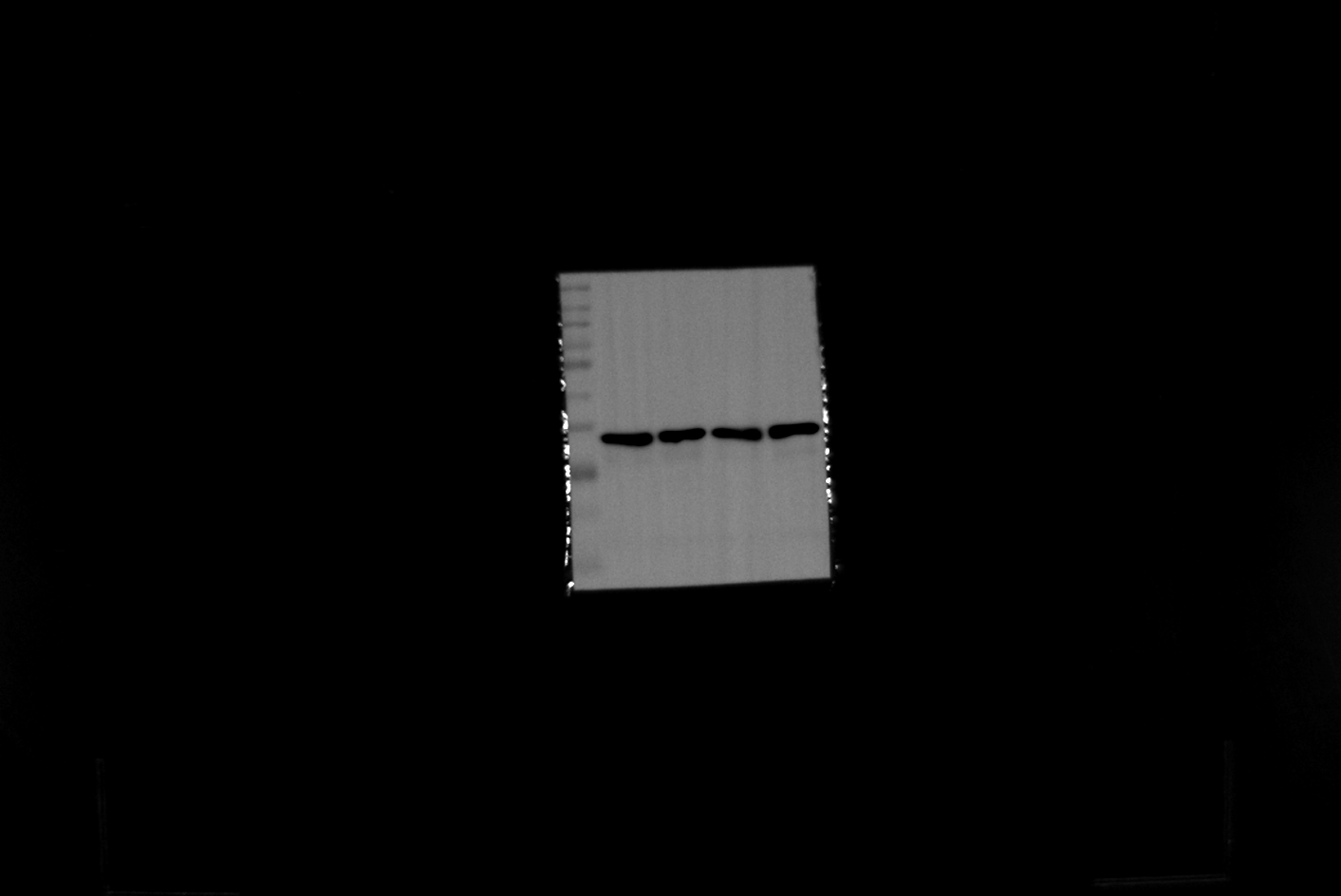


GAPDH 37kDa

Figure 4 A


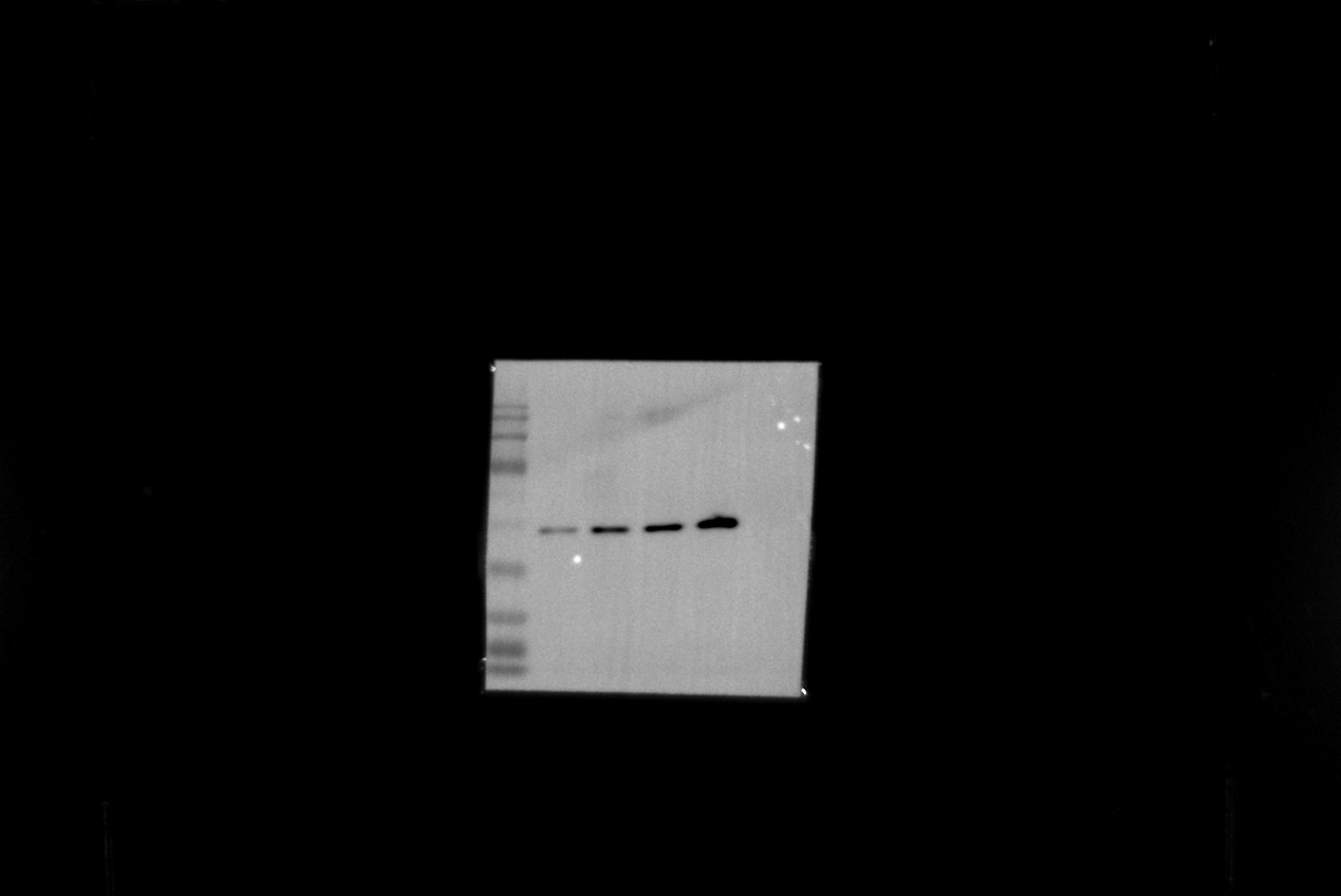


PD-L1 40kDa

Figure 4 A


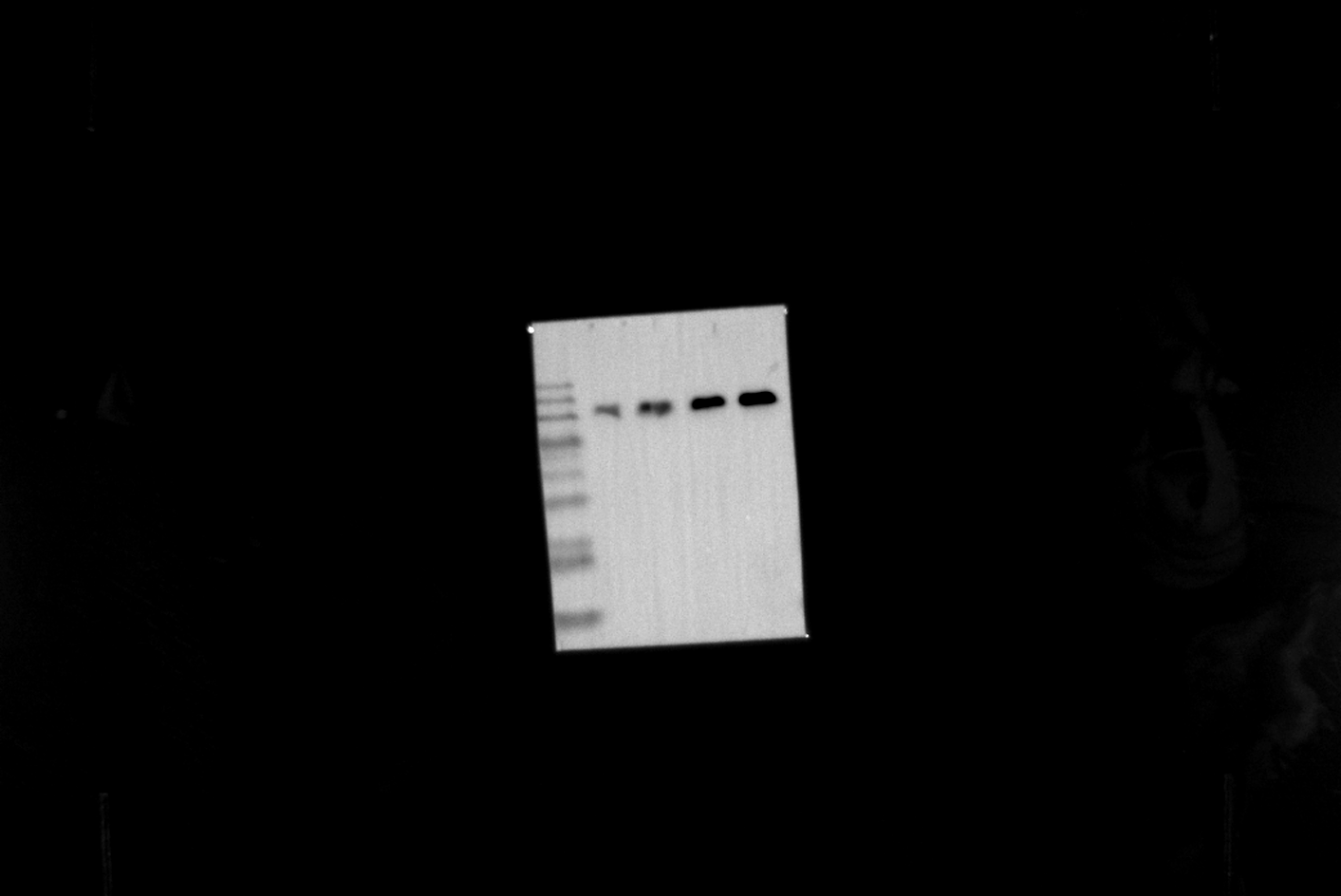


LDLR 115kDa

Figure 4 B


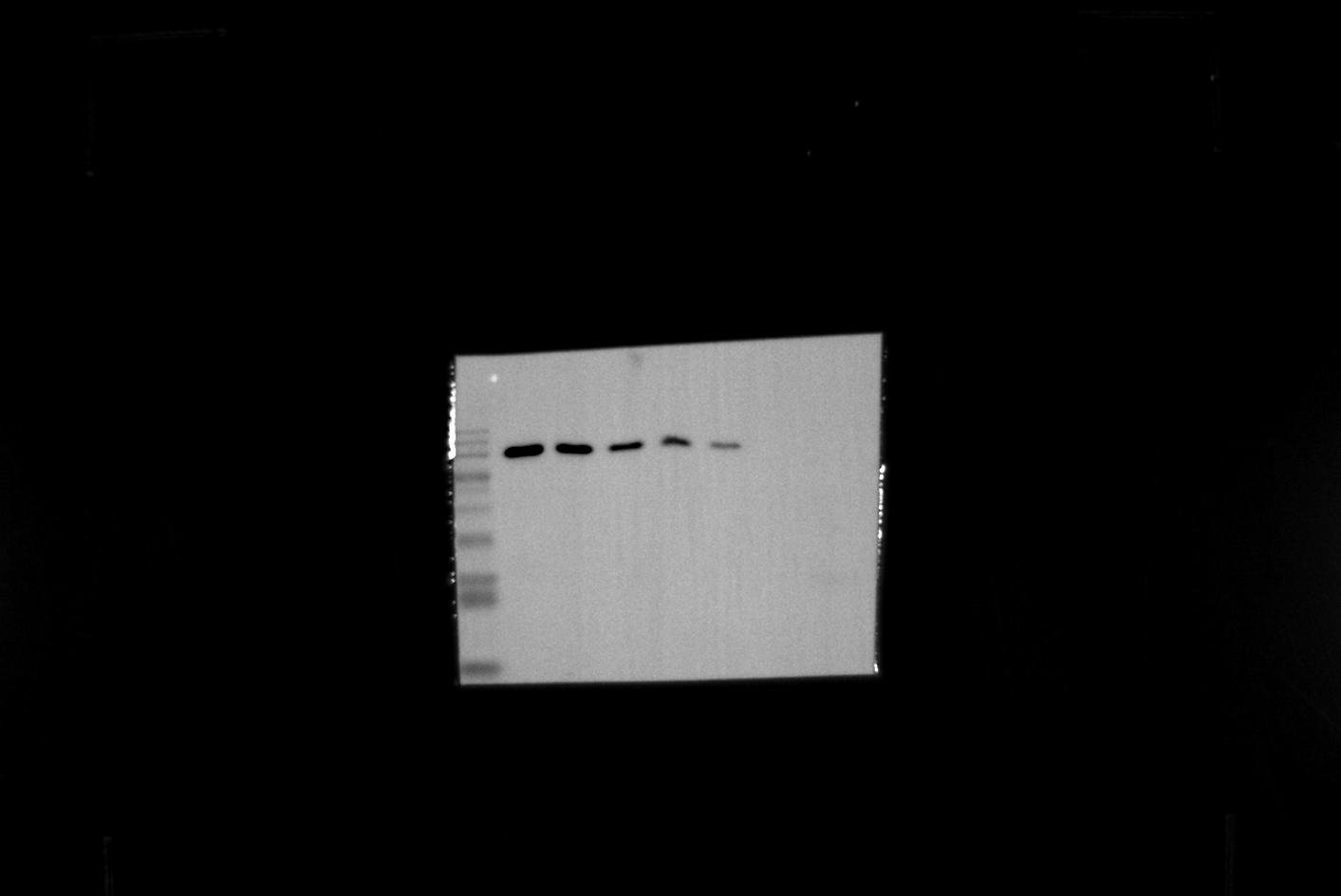


LDLR 115kDa

Figure 4 B


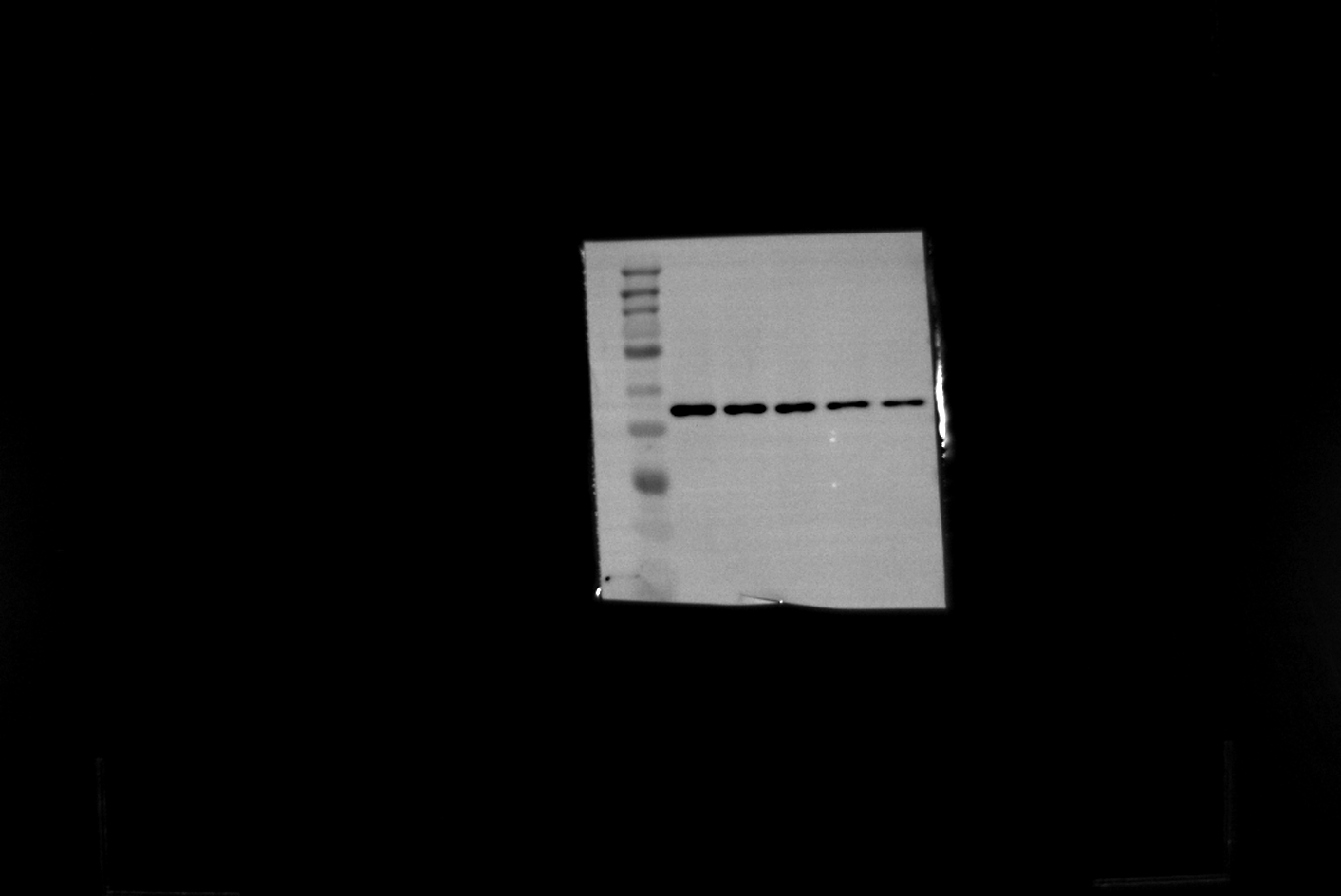


PD-L1 40kDa

Figure 4 B


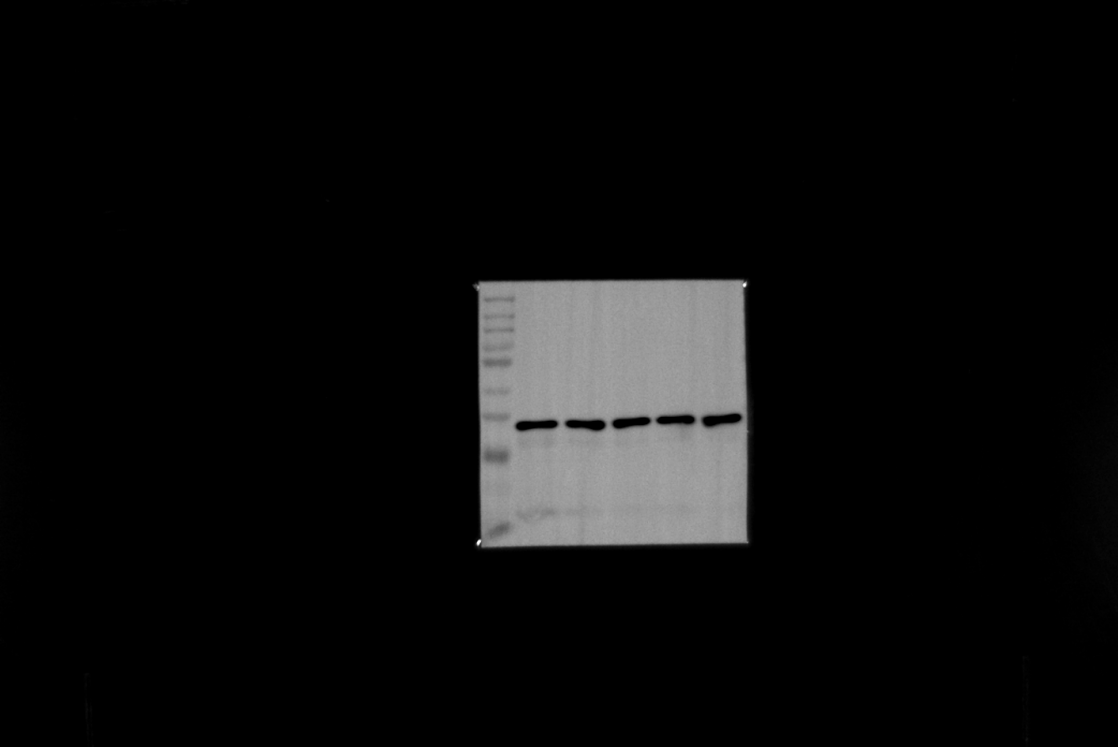


GAPDH 37kDa

Figure 4 C


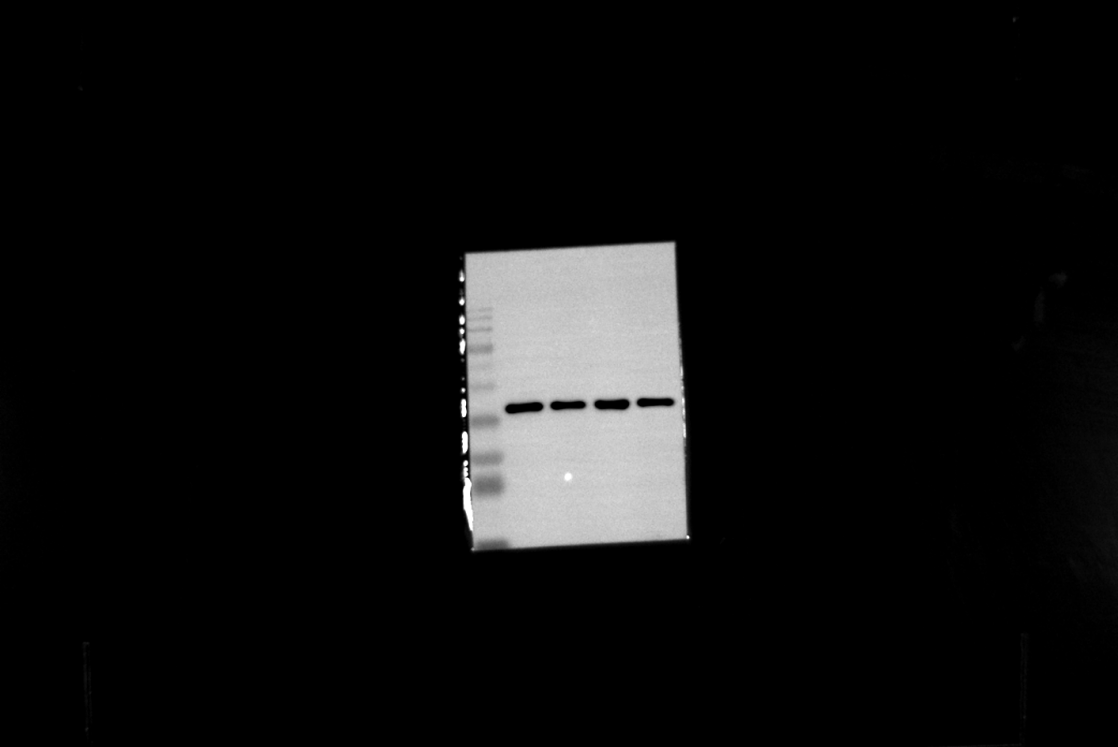


GAPDH 37kDa

Figure 4 C


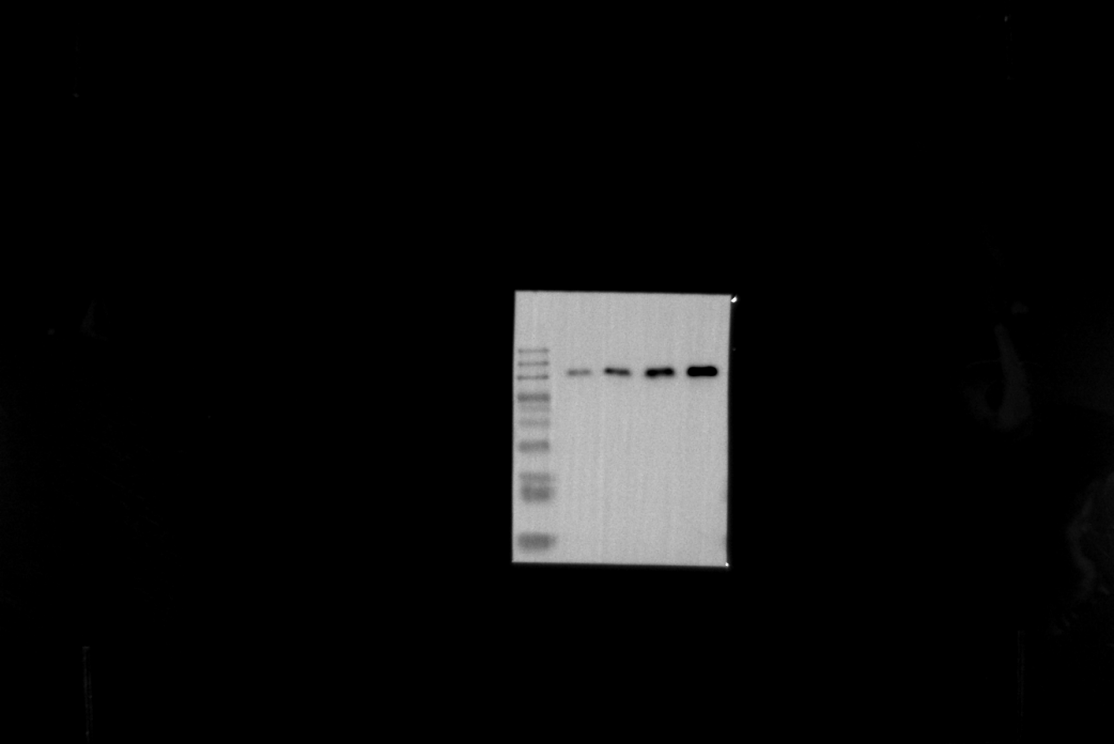


LDLR 115kDa

Figure 4 C


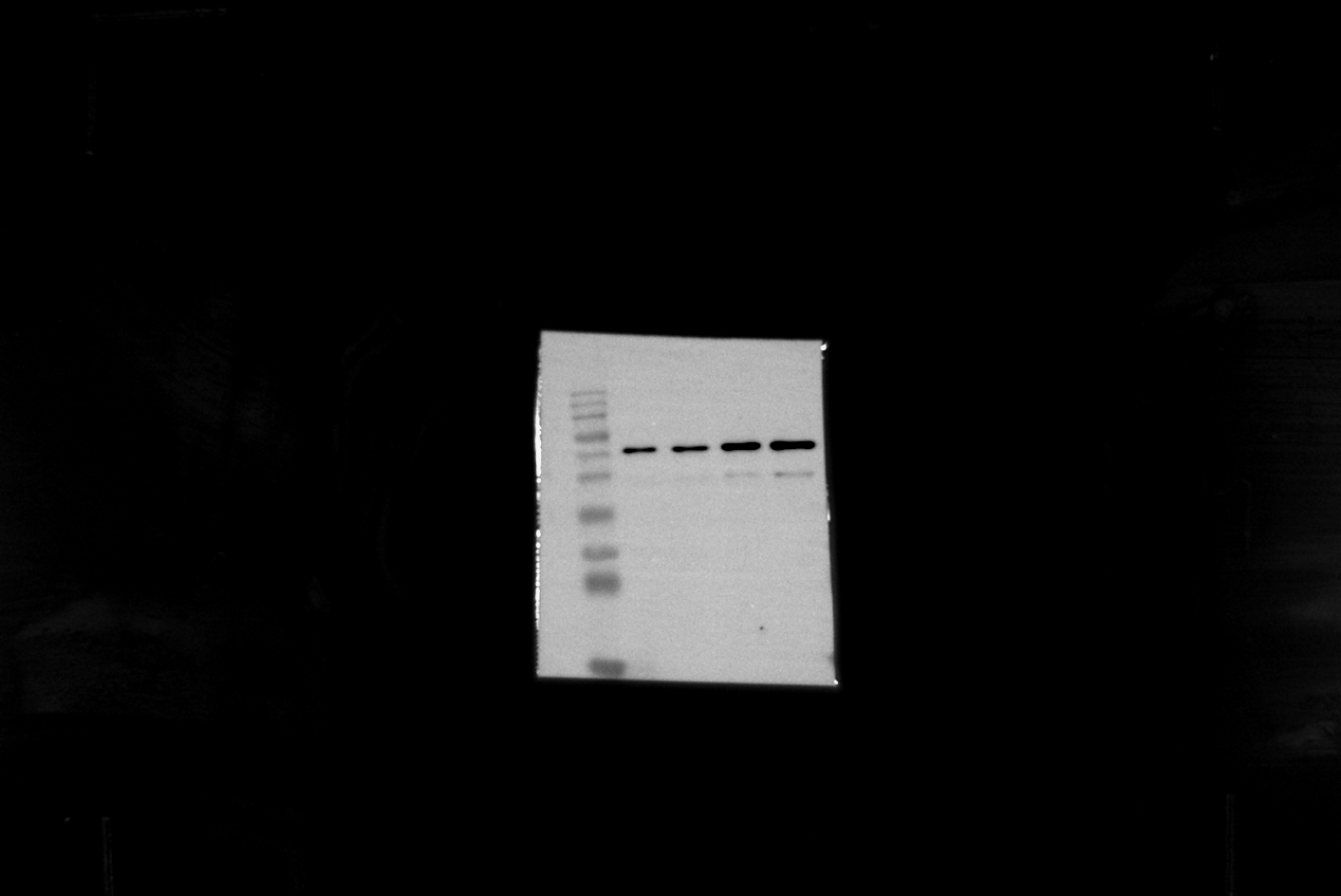


PD-L1 40kDa

Figure 4 D


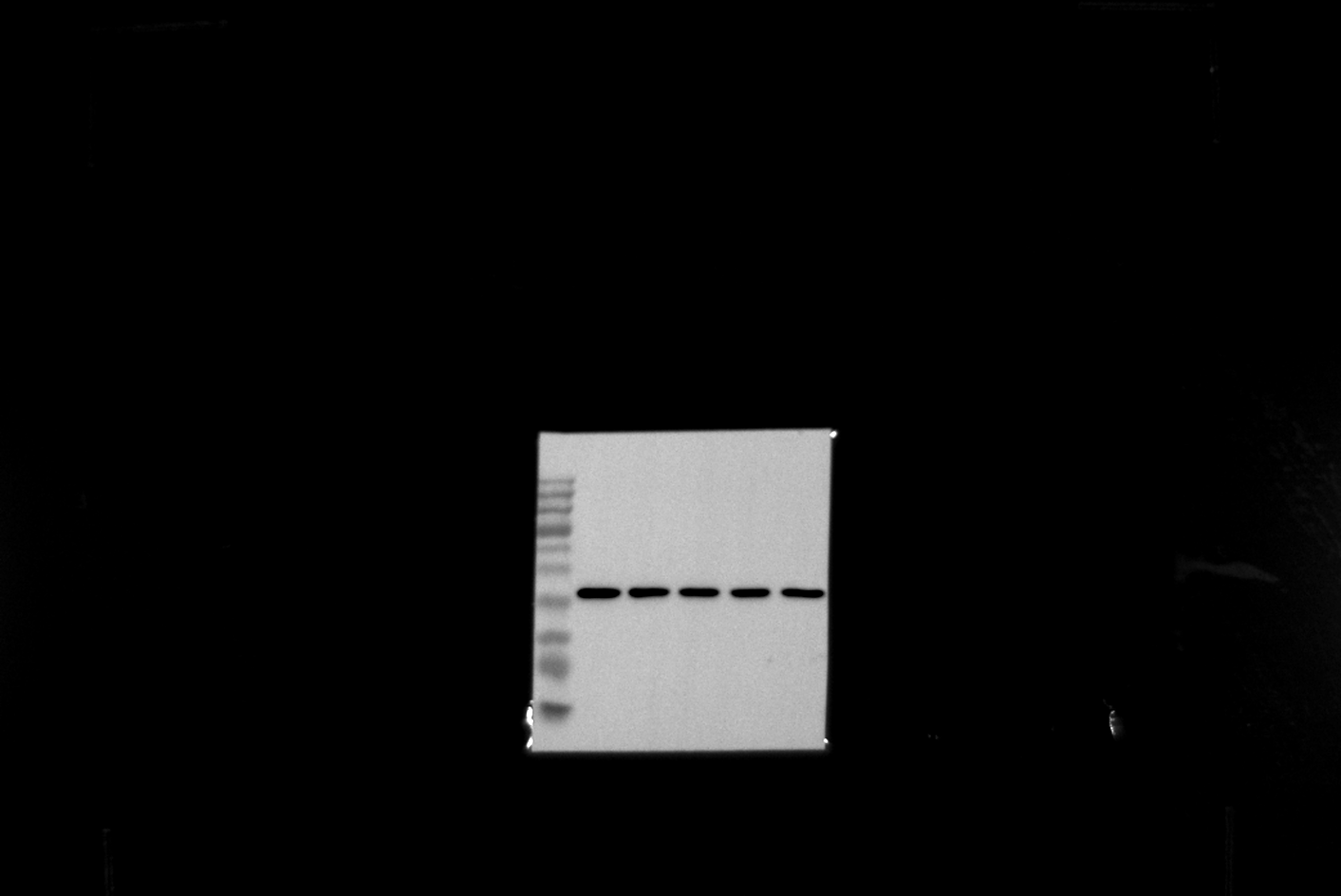


GAPDH 37kDa

Figure 4 D


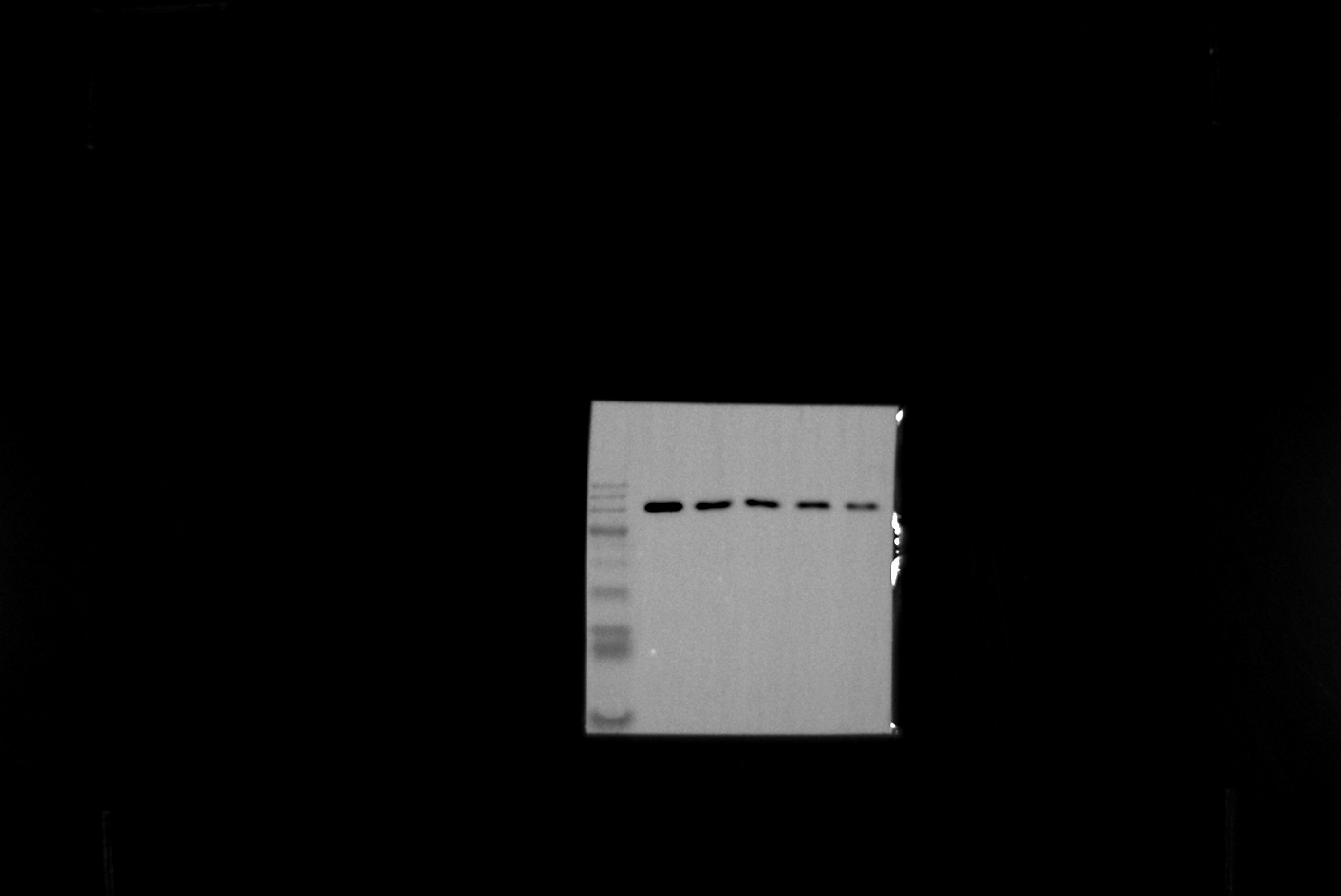


LDLR 115kDa

Figure 4 D


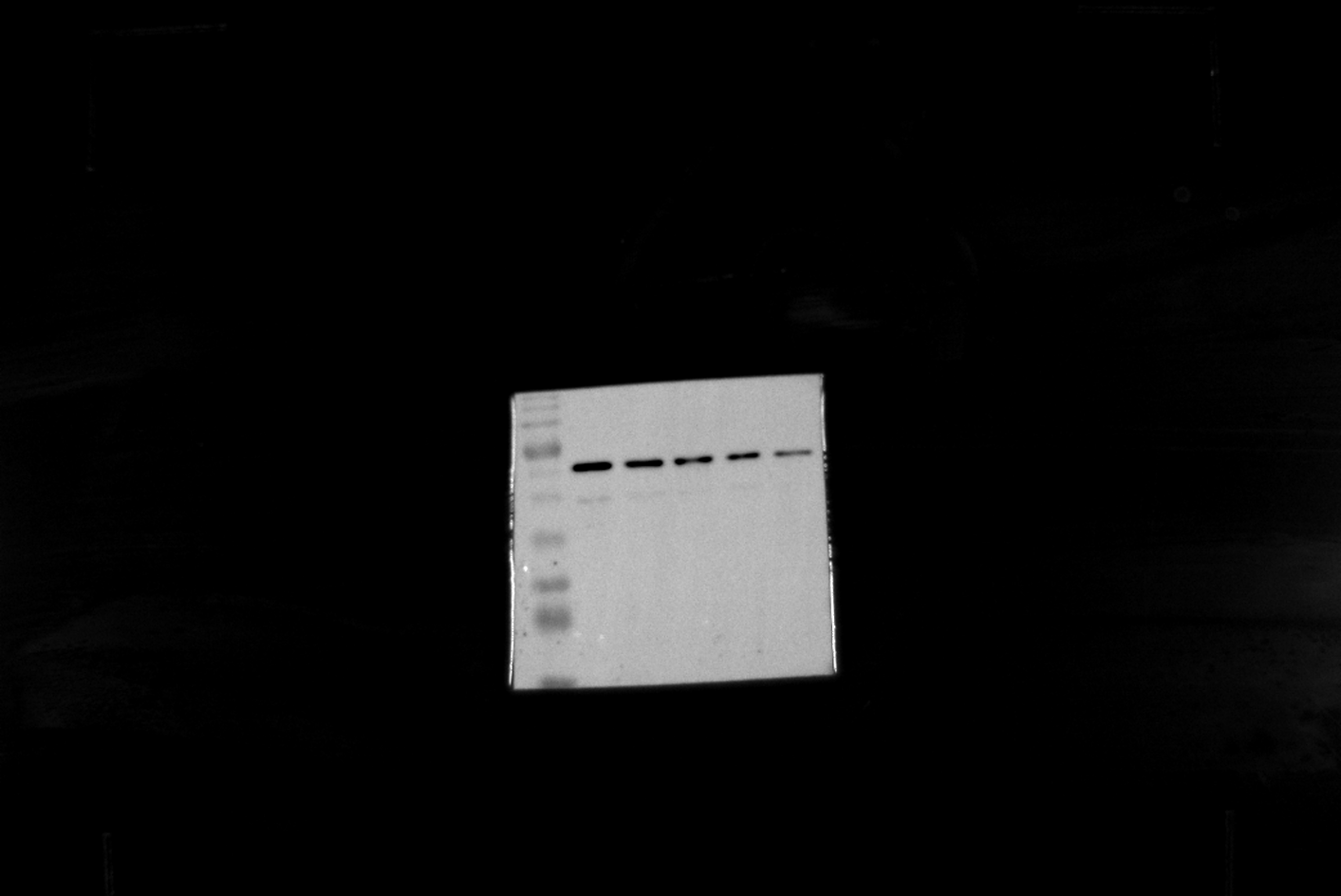


PD-L1 40kDa
